# A novel STAT1 loss-of-function mutation associated with Mendelian susceptibility to mycobacterial disease

**DOI:** 10.3389/fcimb.2022.1002140

**Published:** 2022-10-21

**Authors:** Fanghua Ye, Wen Zhang, Jiajia Dong, Min Peng, Chenying Fan, Wenjun Deng, Hui Zhang, Liangchun Yang

**Affiliations:** Department of Pediatrics, Xiangya Hospital Central South University, Changsha, China

**Keywords:** host genetics, STAT1, MSMD, loss-of-function, IFN-γ

## Abstract

Mendelian susceptibility to mycobacterial diseases (MSMD) is a rare congenital immune deficiency characterized by susceptibility to weakly virulent mycobacteria. Loss-of-function (LOF) mutation of signal transducer and activator of transcription 1 (*STAT1)* is one of the common genetic causes of MSMD. In this study, we identified a patient who presented with multiple lymph node enlargements and multiple osteolytic disruptions. *Mycobacterium gordonae* infection was confirmed by metagenomic next-generation sequencing. Whole-exome sequencing identified a novel paternal heterozygous mutation in exon 22 of *STAT1* (NM_007315.4, c.1892T>C, p.Val631Ala). This variant was confirmed pathogenic by multiple software predictions. Based on functional assays, STAT1 expression in STAT1^V631A^ cells was not different from STAT1^WT^ cells. But STAT1^V631A^ mutation caused much lower activation of STAT1 when stimulated by interferon-γ (IFN-γ). Fluorescence localization analysis revealed that both STAT1^V631A^ and STAT1^WT^ proteins were located in the cytoplasm, and only a few STAT1^V631A^ proteins were translocated to the nucleus in response to IFN-γ. These results suggest that STAT1^V631A^ leads to LOF in IFN-γ-mediated mycobacterial immunity, resulting in MSMD. Treatment with antibiotics has achieved ideal disease control for this patient, and no adverse events occurred during follow-up. The *STAT1* LOF deficiency is a genetic cause of MSMD, which should be considered in patients with mycobacterial disease, especially those with bone involvement.

## Introduction

Host genetics has been proved to play an important role in the susceptibility and severity of infectious diseases, which primarily influence the pathogen invasion and immune response (Ishii et al., 2008). Mendelian susceptibility to mycobacterial diseases (MSMD) known as a primary immunodeficiency disease (PIDs) characterized by susceptibility to weakly virulent mycobacteria such as non-tuberculous mycobacteria, *Bacillus Calmette Guerin (BCG)* and environmental mycobacteria, caused by a single gene mutation (Casanova and Abel, 2002; Bustamante et al., 2014). Presently, multiple genes have been identified, including X-linked (*CYBB* and *NEMO*) and autosomal genes (*IFNGR1*, *IFNGR2*, *IL12B*, *IL12RB1*, etc.), which are involved in the production or response to interferon-γ (IFN-γ) (Rosain et al., 2019; Ying et al., 2019; Bustamante, 2020; Mirzalieva et al., 2022).

Signal transducer and activator of transcription 1 (*STAT1)* belongs to the transcription factor *STAT* family, which is located on chromosome 2 and consists of 25 exons, contains the N-terminal domain, coiled-coil domain, DNA-binding domain, connection domain, SH2 domain, tail segment domain, and trans-activator domain (Horvath, 2000; Ihle, 2001). STAT1 is primarily activated by IFN in the response of the antimicrobial immune system. IFN-γ stimulation leads to STAT1 phosphorylation at the p.Tyr701 site, inducing its homodimerization to form γ-activating factor (GAF), which is translocated to the nucleus. GAF binds to γ-activating sequences to induce transcription of target genes to activate the antimicrobial immune response, followed by STAT1 dephosphorylation back to the cytoplasm (Vinkemeier, 2004). In contrast, IFN-α/β stimulation triggers STAT1 and STAT2 phosphorylation, leading to the formation of the interferon-stimulated genes factor-3 complex, which recognizes IFN-stimulated response element motifs in target genes and involves in the antiviral immune response (Dupuis et al., 2003).

Partial or complete autosomal recessive (AR) *STAT1* deficiency disrupts the IFN-α/β and IFN-γ signaling pathways, which predisposes such patients to weakly virulent intracellular bacterial and viral infections (Immunodeficiency 31B, OMIM #613796). In contrast, autosomal dominant (AD) *STAT1* deficiency has less of an impact on the anti-infective function of the IFN-α/β signaling pathways, and such patients may typically present with only *mycobacterial* infections (Immunodeficiency 31A, OMIM #614892). Intriguingly, the AD gain-of-function (GOF) *STAT1* mutations may contribute to the auto-inflammatory response by enhancing the IFN-a/ß signaling pathway. This enhancement would inhibit interleukin (IL)-17-producing T-cell production and may develop chronic mucocutaneous candidiasis (Immunodeficiency 31C, OMIM # 614162) (Liu et al., 2011). Based on recent studies, loss-of-function (LOF) mutation of *STAT1* is a genetic cause of MSMD that includes AD *STAT1* deficiency and AR *STAT1* deficiency, resulting in isolated MSMD or syndromic MSMD phenotype (Bustamante, 2020). Hence, genetic analysis and the pathogenic function of mutant genes are thus important to research.

In this study, we identified a novel paternal heterozygous *STAT1* mutation (NM_007315.4, c.1892T>C, p.Val631Ala) in a patient with disseminated mycobacterial infection. Functional experiments verified that STAT1^V631A^ provoked lessened STAT1 nuclear translocation and phosphorylation in response to IFN-γ, ultimately leading to MSMD. The relevant literature was also reviewed and analyzed, which expanded our knowledge of the role of *STAT1* as a host genetic factor in mycobacterial infection, and provided an experience for clinicians and patients.

## Materials and methods

### Patient

A female infant was diagnosed with disseminated mycobacterial infections in our hospital. After written informed consent was obtained from the parents, peripheral blood samples were collected from the proband and her healthy parents. Clinical case history, the laboratory, and image results were reviewed. She was treated and followed up regularly.

### Whole-exome sequencing and sanger sequencing

Genomic DNA was extracted from the peripheral blood of the patient and her family. WES, based on NovaSeq 6000 technology sequencing platform, using IDT xGen Exome Research Panel for a capture library building, double-end (Paired-End) sequencing strategy. Raw data>10G, Q30≥80%. Compared with the human genome information in the Single Nucleotide Polymorphism Database, the Human Gene Mutation Database (HGMD), and Exome Sequencing Project 6500 databases according to the American College of Medical Genetics and Genomics (ACMG) guidelines, and screened for suspicious variants. Sanger sequencing was performed on the ABI 3500 Dx platform to verify the WES results.

### Structure and function prediction of mutant proteins

The tertiary structure of the STAT1 protein was predicted by AlphaFold^1^. The SH2 domain of STAT1 in this model has ideal confidence. All structure models were visualized using the molecular graphics program PyMol 2.1. The STAT1^V631A^ structure was mutated by the PyMol mutagenesis tool. The electrostatic potential was calculated and gradient colored using the PyMol vacuum electrostatic program. The potential pathogenicity of the identified variants was predicted by multiple predictive tools such as PolyPhen2.2^2^, SIFT^3^, and Mutation Taster^4^. Mutation Assessor^5^, REVEL^6^.

### Plasmid constructions

The human *STAT1* coding sequence was cloned using reverse transcription PCR. The sequence of primer, forward: 5’-TTAAGCTTGGTACCGAGCTCGCCACCATGTCTCAGTGGTACG-3’; reverse: 5’-CCCTTGCTCACCATGGATCCGCTTCCCCCTCCTCCTACTGTGTTCATCATACTG

TCGAAT-3’. PCR products were ligated into the pcDNA3.1 vector between the *kpnI* and *BamHI* restriction sites using ClonExpress II One Step Cloning Kit (Vazyme, China). Mutations were introduced using Mut Express II Fast Mutagenesis Kit V2 (Vazyme, China) following the manufacturer’s instructions. Flag and GFP were tagged at the C-terminus of the expression plasmid. The constructed plasmids were verified by sequencing.

### Western blot

Human embryonic kidney 293T (HEK293T) cells were transfected using Lipofectamine 2000 (Thermo Fisher, USA) at a 1:1 ratio with either the WT or mutant plasmid. IFN-γ (10^5^ IU/mL, Peprotech, USA) or IFN-α (10^5^ IU/mL, MCE, USA) was used to stimulate the transfected cells for 30 or 60 minutes, or normal saline was used as a control. Western blot analysis was used to assess GAPDH, total STAT1 protein, and phospho-STAT1 (Y701). The primary antibodies used here included mouse anti-Flag (1:2000, Sigma, F1804), rabbit anti-STAT1 (phospho Y701) (1:1000, Abcam, ab109457), and rabbit anti-GAPDH (1:10000, Abcam, ab181602) antibody. The secondary antibodies were peroxidase-conjugated goat anti-rabbit IgG (1:10000, Jackson, #111-035-003) and HRP conjugated Goat Anti-Mouse IgG (1:5000, Servicebio, GB23301).

### Fluorescence microscopy

HEK293T cells were transfected with the WT or mutant plasmid. IFN-γ (10^5^ IU/mL) or IFN-α (10^5^ IU/mL) was used to stimulate the transfected cells for 60 minutes, or normal saline was used as a control. Then fixed with 4% paraformaldehyde and permeabilized with 0.3% Triton X-100 in PBS. After 5% Goat serum Albumin for 1 h, The nuclei were stained with 4,6-diamidino-2-phenylindole (Beyotime, China). Sublocalization of STAT1 was acquired by a confocal microscope (Zeiss, Germany).

### Literature review

Literature searches were conducted in PubMed and Web of Science library databases to find relevant articles published through May 1, 2022, to identify case studies reporting STAT1 LOF deficiency. Functional analysis confirmed the pathogenicity of mutation. Search themes include “STAT1” or “signaling sensor and transcriptional activator 1”, “loss-of-function” or “LOF”, “negative mutation” or “STAT1 deficiency”. All selected articles were reviewed for full text.

## Results

### Clinical features of the patient

**Figure 1:**
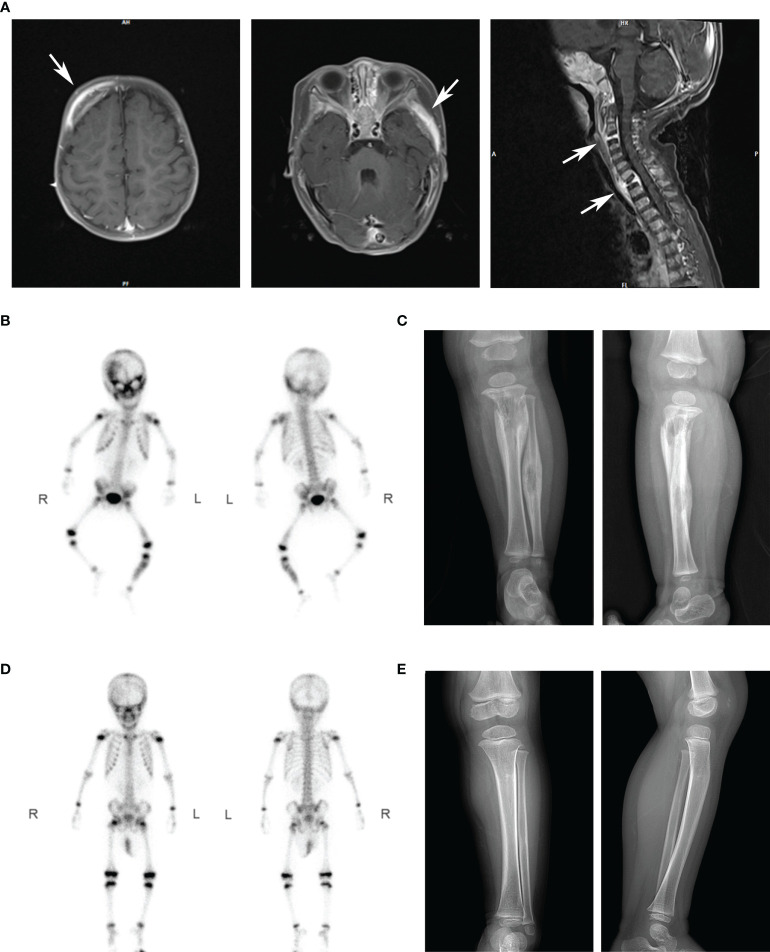
Imaging results of our patients in different periods. **(A)** The MRI scanning showed multiple osteolytic processes in the frontal temporal bone (left), pterygoid bone (middle), and C3 and T1 vertebrae (right). Arrows marked the diseased region. **(B)** After intravenous injection of 99mTc-MDP for 4 hours, anterior and posterior whole-body bone imaging was performed. Results showed abnormal concentration area of imaging agent distribution in multiple skulls (right frontal bone, bilateral orbital bone, sieve bone, pterygoid bone, maxillary bone, occipital bone, and temporal bone), right posterior ribs 10 and 11, left lower femur, and left middle and upper tibiofibular. **(C)** X-ray showed osteolytic changes and periosteal hyperplasia in the upper left tibia and middle left fibula (positive and lateral positions). **(D)** After antibiotics treatment, the abnormal bone metabolism area in the whole-body bone scan was significantly smaller than that before. **(E)** X-ray showed that the range of osteolytic damage was significantly reduced.

The proband was born as the first child uneventfully in a healthy non-consanguineous Chinese family. She was vaccinated with BCG after birth, without adverse reactions. There is no family history of recurrent infection, neoplastic disease, or any known genetic disorder. Her clinical case history is shown in **Table 1**. She presented with intermittent fever, multiple lymph node enlargements, and multiple osteolytic disruptions during her 1st year of life (**Figure 1**). High white blood cell and neutrophil counts were recorded in routine blood tests. The hematologic neoplastic disease was not confirmed because neither bone biopsy nor bone marrow biopsy uncovered tumor cells. Histological analysis of the left tibia revealed chronic inflammation. Nontuberculous mycobacteria DNA was detected by metagenomics next generation sequencing (mNGS) of biopsy tissue, and the strain was identified as *Mycobacterium gordonae*. Her condition continued to improve after treatment with linezolid, isoniazid, and rifampin.

**Table 1:** Clinical case history and treatment process of our patient.

	Visiting hospital age			
	1 year (Hospitalized in another hospital)	1 year (Hospitalized in our hospital)The patient received a positive genetic diagnosis with a variant of *STAT1*.	1 year 6 months(follow-up outpatient)	2 years 2 months(follow-up outpatient)
Chief complaint	Fever, enlarged left submandibular lymph node	Enlarged left submandibular lymph node	–	–
Clinical examinations	Laboratory testing: elevated WBC at 21.51×10^9^/L, NE at 12.62×10^9^/L; increased CRP at 184.39mg/L. B-ultrasound: multiple enlarged lymph nodes in bilateral submandibular and axillae CT head scan: Osteolytic destruction in the frontal bone and alisphenoid bone.	Laboratory testing: Immunoglobulin and lymphocyte subpopulation were normal. HIV serum test, T-SPOT test, G/GM test, bone marrow biopsy, blood culture, and bone biopsy tissue culture and acid-fast staining were all negative. MRI: Bone destruction changes of right frontal bone, sphenoid wing bone, C3 and T1 vertebral bodies (**Figure 1A**). Whole-Body Bone Scan: multiple areas of abnormally elevated bone metabolism (**Figure 1B**). X-ray of the left tibiofibula: dissolved bone change, periosteal proliferation (**Figure 1C**). CT of the chest, abdomen and pelvis: normal Left tibia biopsy: chronic inflammation. Bone biopsy tissue mNGS: positive for Mycobacterium gordonae.	Laboratory testing: Normal CBC and blood Biochemistry. B-ultrasound: superficial lymph nodes are normal. X-ray of the left tibiofibula: the damage range of bone dissolution was significantly reduced.	Laboratory testing: Normal CBC and blood Biochemistry. Whole-Body Bone Scan: recovery of abnormal bone metabolism area. (**Figure 1D**). X-ray of the left tibiofibula: normal (**Figure 1E**).
Clinical diagnosis	Lymphnoditis, unexplained bone damage	MSMD, Lymphnoditis, Multifocal osteomyelitis	MSMD, Multifocal osteomyelitis (recovery phase)	
Treatments	Cefoperazone, support treatment including antipyretic, rehydration.	Linezolid, isoniazid and rifampicin	Consolidation treatment of isoniazid and rifampicin.	
Outcomes	No further fever	Lymph nodes gradually shrink	The patient’s overall condition was stable without adverse events.	

CT, Computer Tomography; MRI: Magnetic resonance imaging; WBC, White Blood Cell; NE, neutrophilicgranulocyte; CRP, C-reactive protein; mNGS, metagenomic next-generation sequencing; MSMD, mendelian susceptibility to mycobacterial diseases; CBC, complete blood count.

### Molecular genetic analysis of the proband

**Figure 2:**
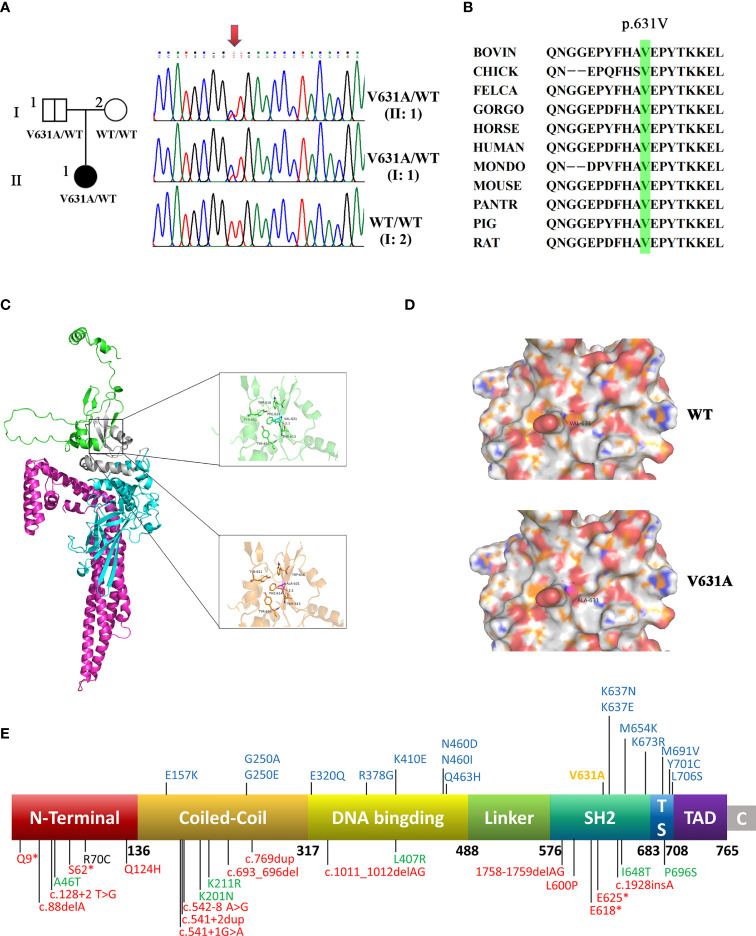
A novel heterozygous mutation was identified in *STAT1*. **(A)** The family pedigree. DNA sequence chromatograms of the patient and her family. Arrows marked the mutated site. c.1892T>C in exon 22 of *STAT1* caused a conservative substitution of valine to alanine acid (p.V631A). **(B)** V631 amino acids in STAT1 protein are highly conserved during evolution. **(C)** The crystal structure of the STAT1 is shown in cartoon representation and colored for the coiled-coil domain (red), DNA-binding domain (cyan), linker domain (grey), and SH2 domain (green). Magnified pictures displayed the Val-631 (top), and Ala-631 (bottom). The R group of Ala-631 is methyl, which is less hydrophobic than the dimethyl group of Val-631, so the hydrophobic interaction with the surrounding hydrophobic amino acids will be weakened to a degree. **(D)** The calculated electrostatic potentials of STAT1^WT^ and STAT1^V631A^ are mapped on the surface and colored in a gradient from red (negative) to blue (positive). There is no significant change after mutation. **(E)** Domains of *STAT1* protein: the coiled-coil domain (CCD), DNA-binding domain (DBD), linker domain (LD), SH2 domain (SH2D), tail segment domain (TSD), and trans-activator domain (TAD). AR forms of complete (red) and partial (green) *STAT1* deficiency are shown below the protein. AD *STAT1* LOF mutations are shown above the protein with blue (previous study) and yellow (current study).

According to the age of onset and clinical phenotype, innate immune abnormalities were considered for the patient. Therefore, the whole-exome sequencing was performed for the patient and her parents. A novel paternal heterozygous variant in the *STAT1* gene (NM_007315.4: c.1892T>C, p. Val631Ala) was identified, which was confirmed by Sanger sequencing (**Figure 2A**). Her healthy father was also found to be heterozygous for the p.V631A mutation, suggesting that this variant with low clinical penetrance, like the previously reported p.L706S and p.Q463H (Dupuis et al., 2001; Chapgier et al., 2006). The mutation was not found in the gnomAD, ClinVar, or HGMD. This mutation was located within the SH2 domain, resulting in a valine to alanine substitution at position 631. The alanine residue was not observed at this position in homologous sequences, suggesting high conservation among different species (**Figure 2B**).

### Prediction and analysis of protein bioinformatics

Val-631 can form strong hydrophobic interactions with amino acid residues such as Trp-616, Tyr-651, Tyr-634, and Thr-613, which play a crucial role in stabilizing the protein. The mutant Ala-631 is less hydrophobic than Val-631, and the hydrophobic interaction with the surrounding hydrophobic amino acids will be weakened to a degree (**Figure 2C**). Both amino acids are hydrophobic, so there is no significant change on the electrostatic surface (**Figure 2D**).

*STAT1^V631A^* mutation was classified as a “variant of uncertain significance” (PM2_Supporting + PP2 + PP3) according to the 2015 ACMG guidelines (Richards et al., 2015). Prediction of protein function by bioinformatics software was performed. It was considered as “probably damaging” with PolyPhen2, “deleterious” with SIFT, “disease-causing automatic” with Mutation Taster, and “Medium” with Mutation Assessor. The REVEL score was 0.856. These results strongly indicated that the mutation might alter the STAT1 function.

### Functional consequences of p.V631A variant

**Figure 3:**
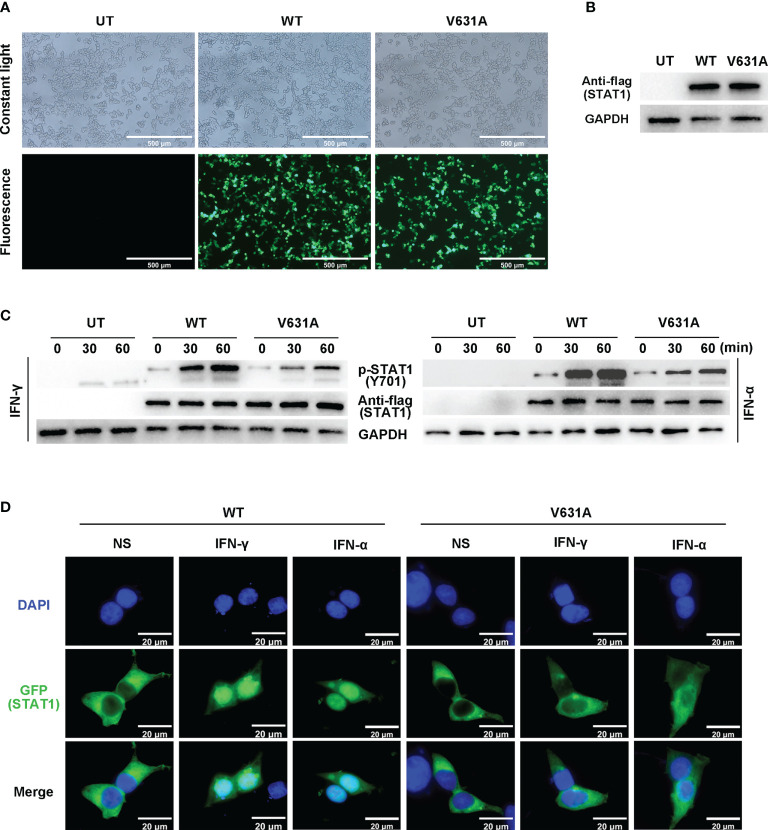
Functional assays of STAT1^WT^ and STAT1^V631A^. **(A)** The cell growth diagram after transfection showed that there was no significant difference in GFP transfection efficiency among WT and V631A groups. UT, untreated. **(B, C)** Western blot shows the STAT1 expression **(B)** and phosphorylation **(C)** of STAT1 in the human embryonic kidney 293T (HEK293T) cells transfected with STAT1 constructs. The total STAT1 and GAPPDH were used as the loading controls. One experiment representative of three independent experiments is shown. **(D)** Localization of GFP-labeled STAT1 under non-stimulated (NS), IFN-γ stimulated (10^5^ IU/mL, 60 mins), or IFN-α stimulated (10^5^ IU/mL).

We performed functional validation by transfecting either flag-tagged WT or mutant constructs into HEK293T cells. According to the intracellular fluorescence signals, the transfection efficiency of WT and mutant groups were comparable (**Figures 3A, B**). Western blot showed the total protein expression level of STAT1 was not different between STAT1^WT^ and STAT1^V631A^ cells (**Figures 3B, C**), which is consistent with the results of bioinformatics analysis of minor structural changes after mutation. The phosphorylated STAT1 of STAT1^V631A^ cells was significantly lower than STAT1^WT^ cells after IFN-γ and IFN-α stimulation (**Figure 3C**). The localization of STAT1 protein was detected by fluorescence microscopy. Before stimulation, the STAT1 proteins were located in the cytoplasm in both STAT1^WT^ cells and STAT1^V631A^ cells. After IFN-γ and IFN-α stimulation, most of the STAT1 was translocated to the nucleus in STAT1^WT^ cells, but only a few STAT1 were translocated to the nucleus in STAT1^V631A^ cells (**Figure 3D**). Collectively, these results suggested that the STAT1^V631A^ represented a LOF mutation by weakening the phosphorylation and nuclear accumulation of STAT1.

### Literature review of STAT1 LOF deficiency patients

Here, we summarized the clinical features and genetic characteristics of symptomatic patients with *STAT1* LOF deficiency in previous study (**Table 2**; **Figure 2E**, details in **Supplementary 1**). A total of 64 patients (31 AD *STAT1* deficiency and 33 AR *STAT1* deficiency) were selected for retrospective analysis (Dupuis et al., 2001; Chapgier et al., 2006; Sampaio et al., 2012; Tsumura et al., 2012; Hirata et al., 2013; Muriel-Vizcaino et al., 2016; Boudjemaa et al., 2017; Kagawa et al., 2017; Ueki et al., 2017; Ying et al., 2019; Bhattad et al., 2021; Le Voyer et al., 2021; Liu et al., 2022; Zhou et al., 2022). Clinical data for one patient were not available (Muriel-Vizcaino et al., 2016). Thirty-nine mutations have been reported and functionally validated, which were located throughout the STAT1 protein (**Figure 2E**), including 25 missense, four deletions, two insertion, four nonsense, and four splicing mutations.

**Table 2:** Demographic and infectious phenotypes of the international cohort of patients with STAT1 LOF deficiency.

Characteristic, n (%)	All Patients (N=63)	AD STAT1 deficiency (N=31)	AR STAT1 deficiency (N=32)	*P* value
Gender				
Male	28 (44.4)	13 (41.9)	15 (46.9)	
Female	35 (55.6)	18 (58.1)	17 (53.1)	0.693^a^
Age of onset				
<1 yr	40 (63.5)	11 (35.5)	29 (90.6)	
≥1 yr	23 (36.5)	20 (64.5)	3 (9.4)	<0.001^b^
Mycobacterial diseases	55 (87.3)	31 (100)	24 (75)	
isolated MSMD^*^	36 (57.1)	28 (90.3)	8 (25)	
Syndromic MSMD^#^	19 (30.2)	3 (9.7)	16 (50)	<0.001^b^
Site of mycobacterial diseases				
Disseminated	28 (44.4)	17 (54.8)	11 (34.4)	
Bone	25 (39.7)	20 (64.5)	5 (15.6)	
Lymph node	22 (34.9)	8 (25.8)	14 (43.8)	
Skin	26 (41.3)	18 (68.1)	8 (25)	
Lung	10 (15.9)	4 (12.9)	6 (18.8)	
Liver/spleen	11 (17.5)	1 (3.2)	10 (31.2)	
CNS	2 (3.2)	1 (3.2)	1 (3.1)	
pathogen spectrum				
*M. bovis-BCG*	34 (54)	20 (64.5)	14 (43.8)	
*M. avium*	7 (11.1)	4 (12.9)	3 (9.4)	
*M. tuberculosis*	7 (11.1)	7 (22.6)	0	
*M. kansasii*	2 (3.2)	0	2 (6.3)	
*M. abscessus*	1 (1.6)	0	1 (3.1)	
*M. szulgai*	1 (1.6)	0	1 (3.1)	
*M. scrofulaceum*	1 (1.6)	0	1 (3.1)	
*M. malmoense*	1 (1.6)	0	1 (3.1)	
*Undefined species*	3 (4.8)	1 (3.2)	2 (6.3)	
Viral infections	21 (33.3)	3 (9.7)	18 (56.3)	<0.001^b^
Other bacterial infections	8 (12.7)	0	8 (25)	0.05^c^
Fungal Infection	7 (11.1)	0	7 (21.9)	0.011^c^

MSMD, mendelian susceptibility to mycobacterial diseases; AD, autosomal dominant; AR, autosomal recessive. CNS, central nervous system. ^*^ Isolated MSMD is characterized by a selective predisposition to one or a few infectious agents. ^#^ Syndromic MSMD is defined as a composite of a Mycobacterium infection with another common infection phenotype (e.g. type I interferonopathy). ^a^ chi-square test; ^b^ Correction chi-square test; ^c^ fisher exact test.

AD *STAT1* deficiency manifests frequently as MSMD, virus infection in three cases, and bacterial or fungal infection in none. Some individuals with AD *STAT1* deficiency are asymptomatic. In contrast, patients with AR *STAT1* deficiency present with infections at a younger age and more often present with syndromic MSMD caused by other bacteria, viruses and fungi. MSMD in *STAT1* LOF patients was most frequently caused by *M. bovis-BCG* (54%). The mycobacterial involved disseminated (44.4%), skin (41.3%), bone (39.7%), lymph node (34.9%), liver/spleen (17.5%), lung (15.9%), and central nervous system (3.2%) (**Table 2**).

Most patients with isolated MSMD were treated successfully with anti-mycobacterial therapy. Hepatitis, haemophagocytic lymphohistiocytosis (HLH), and disseminated intravascular coagulation are severe complications of AR *STAT1* deficiency. Besides anti-infectives and antivirals, immunosuppressants and biologics are also used to treat AR *STAT1* deficiency. Twelve patients with AR STAT1 deficiency received HSCT, four died, and none of the eight surviving patients developed viral or mycobacterial infections after transplantation (Le Voyer et al., 2021).

## Discussion

In this research, we identified a novel paternal heterozygous *STAT1* mutation in the SH2 domain (p.V631A) associated with disseminated mycobacterial infection. Clinical features and functional studies confirm the loss of function of STAT1^V631A^ in the IFN-γ pathway, leading to host susceptibility to mycobacterial disease. This is an enrichment of the clinical and molecular phenotypic studies of *STAT1*.

The SH2 domain (residues 577-683) is required for the recruitment of STAT1 to activated interferon receptors and plays an important role in Tyr701 phosphorylation. Phosphorylation-activated STAT1 forms a dimer that enters the nucleus and triggers high-affinity DNA binding and gene transcription (Boisson-Dupuis et al., 2012). Our study showed that V631A mutation reduced STAT1 phosphorylation and nuclear accumulation, consistent with other LOF heterozygous mutations located in the SH2 structural domain (e.g. K637E, K673R, and M654K) (Sampaio et al., 2012; Tsumura et al., 2012). Of four possible STAT1 dimers (WT/WT, WT/V631A, V631A/WT, V631A/V631A), only WT homodimers (WT/WT) formed normal, high-affinity phosphate dimers, which could enter the nucleus and participate in downstream signaling (Dupuis et al., 2001). In addition to the SH2 domain, STAT1 also includes DNA binding domain (residues 318-488) that mediate DNA binding and transcriptional activity, coil-coil domain (residues 137-317) that mediate the interaction between proteins and plays an important role in the formation and dephosphorylation of STAT1 dimer, tail segment domain (residues 684-708) that contain the key amino acid (Tyr701) phosphorylated by JAK kinase (Lim and Cao, 2006; Mao et al., 2005). The association of the variations with the phenotypes in these domains requires further investigation.

The genetic etiological diagnosis of MSMD relies primarily on NGS tests. Reduced cellular response to IFN-γ as a result of *STAT1* deficiency is fundamental to susceptibility to mycobacterial disease. Widely, any STAT1 mutation may contribute to the mycobacterial disease phenotype (Bustamante et al., 2014). Contradictory and mysterious, several investigations have also noted the incidence of mycobacterial disease in patients with GOF of STAT1 (Toubiana et al., 2016). We reviewed the clinical data of patients with *STAT1* LOF deficiency. A total of 36 *STAT1* variants have been reported. With frameshift and nonsense mutations being highly associated with LOF. However, for a novel heterozygous missense mutation, it is difficult to identify whether the functional alteration is GOF or LOF merely from the mutation site and amino acid change. Most patients with autosomal dominant LOF mutations have a partial deletion of STAT1 phosphorylation levels in response to IFN-α or IFN-γ stimulation (Sampaio et al., 2012; Tsumura et al., 2012). However, a minority of patients have normal phosphorylation of STAT1 but exhibit impaired binding activity to DNA (Chapgier et al., 2006). Therefore, functional studies of heterozygous mutants are very important. Alanine scanning mutations established by Kagawa and colleagues are highly sensitive to estimating the function of STAT1 in the discoid and DNA-binding domains, which may be a valuable tool to assess an unknown mutation (Kagawa et al., 2017). However, for other domains or more types of variants, more prediction methods need to be developed.

The phenotypic categories of isolated/syndromic MSMD and genetic functional analysis contribute to our better understanding of the association between the genotype and phenotype (Bustamante, 2020). Our results showed that autosomal dominant LOF of *STAT1* tends to cause isolated MSMD, whereas autosomal recessive *STAT1* is mostly responsible for syndromic MSMD, the latter are commonly comprised of fatal complications. This will facilitate clinicians to manage such patients in a predictive manner. Literature review shows that MSMD occurs in patients with *STAT1* LOF deficiency mainly in infancy, with recessive patients presenting earlier than dominant patients, mostly before 1 year of age. The incidence of multifocal osteomyelitis was high, and some patients even developed osteomyelitis without other organ involvement (Dupuis et al., 2001; Hirata et al., 2013; Boudjemaa et al., 2017; Kagawa et al., 2017). It was found that this may be due to impaired IFN-γ response and increased osteoclast differentiation in patients with *STAT1* deficiency (Tsumura et al., 2022). Two cases of multifocal osteomyelitis had been misdiagnosed as neuroblastoma and Langerhans cell histiocytosis (Boudjemaa et al., 2017; Zhou et al., 2022). Despite histological analysis, another case of multifocal osteomyelitis was suspected sarcoidosis disease (Tsumura et al., 2022). The delay in diagnosis may be related to the low rate of positive cultures for *mycobacteria*. The mNGS method provides rapid, easy, and accurate identification of pathogenic bacteria and improves the detection of mycobacterial disease. Therefore, in clinical practice, *STAT1* LOF deficiency needs to be considered in patients with mycobacterial osteomyelitis at a young age of onset. Molecular diagnostics in MSMD can provide families with genetic counseling and pave the way for a better understanding of treatment options for the pathogenesis of mycobacterial disease. For example, for patients with an incomplete lack of cellular response in *STAT1* deficiency, antibiotics, antiviral agents or recombinant IFN-γ can achieve the desired efficacy (Sampaio et al., 2012; Hirata et al., 2013). In contrast, HSCT is curable in patients with complete *STAT1* deficiency, who need to be alerted to fatal viral infections and serious complications (Naviglio et al., 2017; Le Voyer et al., 2021).

In conclusion, there are few reports about LOF of *STAT1* variants to date. The molecular genetic mechanism of *STAT1* related MSMD remains to be investigated. A study of the genetic background of the host can not only clarify the process of mycobacterial infection and immune response but also guide the prediction of the disease and the development of treatment strategies. Furthermore, with the rapid development of NGS, many new candidate genes and pathways critical for mycobacterial immunity may be identified.

## Data availability statement

The datasets presented in this study can be found in online repositories. The names of the repository/repositories and accession number(s) can be found below: CNGB, CNP0003320.

## Ethics statement

Written informed consent was obtained from the individual(s), and minor(s)’ legal guardian/next of kin, for the publication of any potentially identifiable images or data included in this article. This study was reviewed and approved by the Ethics Committee of Xiangya Hospital of Central South University.

## Author contributions

FY and WZ performed the experiments and analyzed the data. FY wrote the manuscript. WD and HZ screened the relevant literature. JD, MP and CF collected and analyzed the clinical data. LY designed and guided the study and revised the manuscript critically. All authors contributed to the article and approved the submitted version.

## Funding

This work was supported by the National Natural Science Foundation of China (NO.82000137, NO.82002118), and the Natural Science Foundation of Hunan Province (NO.2020JJ4918).

## Acknowledgment

We thank the patient and her parents for their participation in this study. We thank the Medical Research Center of Xiangya Hospital Central South University for providing the platform and the personnel who work there for giving support and guidance.

## Conflict of interest

The authors declare that the research was conducted in the absence of any commercial or financial relationships that could be construed as a potential conflict of interest.

## Publisher’s note

All claims expressed in this article are solely those of the authors and do not necessarily represent those of their affiliated organizations, or those of the publisher, the editors and the reviewers. Any product that may be evaluated in this article, or claim that may be made by its manufacturer, is not guaranteed or endorsed by the publisher.

## Footnotes

1
https://alphafold.ebi.ac.uk/entry/P42224

2
http://genetics.bwh.harvard.edu/pph2/

3
http://sift.jcvi.org/

4
http://mutationtaster.org/

5
http://mutationassessor.org/r3/

6
https://sites.goolgle.com/site/revelgenomics/
